# Directed energy deposition of 18NiM300 steel: effect of process and post processing conditions on microstructure and properties

**DOI:** 10.1080/14686996.2024.2346071

**Published:** 2024-05-20

**Authors:** Stefano Felicioni, Alberta Aversa, Erica Librera, Federica Bondioli, Paolo Fino

**Affiliations:** a Department of Applied Science and Technology, Politecnico di Torino, Torino, Italy; b Consorzio Interuniversitario Nazionale per la Scienza e Tecnologia dei Materiali (INSTM), Firenze, Italy; c Prima Additive, Prima Industrie S.p.A ., Collegno, Italy

**Keywords:** Additive manufacturing, directed energy deposition, steel, 18-Ni M300, inert chamber, austenite reversion, phase transformation, microstructure, mechanical properties

## Abstract

This current study investigates the effect of Direct Energy Deposition (DED) process conditions on the properties and microstructure of M300 maraging steel samples. The investigation centers on two key factors: laser power and deposition environment. The microstructure of this tool steel is analyzed by computing the Primary Cellular Arm Spacing. The findings revealed a significant influence of both inert atmosphere and laser power on cooling conditions. These different cooling rates influence the phase content as demonstrated by X-Ray Diffraction and Electron Backscatter Diffraction measurements. It was demonstrated the presence of different content of residual austenite at cell boundaries. These distinct microstructural features caused variations in the hardness values of the printed samples. Furthermore, a direct aging heat treatment was implemented, that was chosen from Differential Scanning Calorimetry measurements results. This heat treatment proves effective in achieving consistent hardness increases and eliminated the differences among samples built in different process conditions. This outcome suggests the possibility of selecting the most economically viable DED parameters for optimal results.

## IMPACT STATEMENT

This study innovatively explores how DED process conditions impact M300 maraging steel microstructure, revealing crucial insights for optimizing properties and achieving consistent results, ensuring economically viable applications.

## Graphical abstract

**Figure:**
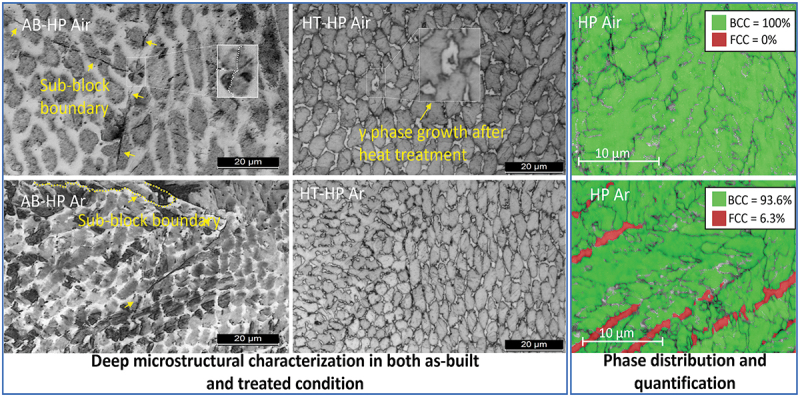

## 1. Introduction

ASTM-F2792 [1] defines Additive Manufacturing (AM) techniques as processes of joining materials to make objects from 3-D model data, usually layer upon layer as opposed to subtractive manufacturing technologies. Direct Energy Deposition (DED) is one of the primary metal AM technologies used to produce near-net-shape large parts with minimum material wastage. In a DED process, the raw metallic material can take the form of powder or wires that are fed above the building surface by single or multiple nozzles and simultaneously molten by a focused energy source. The energy source can be a laser plasma arc or a collimated electron beam.

Many process parameters of powder-based laser DED directly influence the consolidation, the microstructure, and the properties of DED parts. Some of these parameters are laser power, scan speed, hatch spacing, laser beam diameter, process atmosphere. The effects of these parameters can be difficult to identify as the continuous scanning of the parts leads to a complex thermal history. Therefore, the L-DED process can result in a large variety of microstructures and mechanical properties that might also vary along the build direction [2].

In terms of materials, this technique is mostly used with stainless steels. In recent years, however, maraging steels, and in particular 18Ni-300 (M300), have also gained a great deal of interest, mainly due to their combination of high strength and toughness. This makes them suitable for many of the applications that exploit the peculiarities of AM technologies. Sectors in which maraging steels find applications include aerospace and tool manufacturing [3]. These applications often involve complex geometries and limited production quantities; therefore, the use of conventional manufacturing methods can be extremely costly. Furthermore, maraging steels are characterized by an excellent weldability and thus AM processability, owing to their low carbon content (<0.03%) which mitigates carbide formation [4]. The good AM processability of these steels has been amply demonstrated in the literature on Laser Powder Bed Fusion (L-PBF) AM process [5].

Up to now, only few studies have been carried out on the L-DED process of M300 maraging steel. The available studies, that mainly focus on process optimization and defect mitigation, confirm that dense and crack-free samples can easily be built by DED [6,7]. Chen et al. [8] and Félix-Martínez et al. [9], for example, found optimized process parameters using single scan tracks, single layers, and thin walls.

The production of maraging steels components also entails complex heat post-treatment procedures that must be conducted for the improvement of their mechanical performance. These heat treatments can be selected based on the initial production process and on the application of the material, and generally involve austenitization and aging [3] to tailor the austenite content and the precipitation of strengthening phases. As noted by Bai et al., the efficacy of these heat treatments has been extensively proven, particularly in the context of casting and wrought maraging steels [10]. However, given that additive manufacturing techniques lead to unique microstructural outcomes, specific investigations are crucial for effectively controlling the distinctive phase transformations behavior of this steel [11]. It is important to underline that although a significant austenite content is typically unfavorable, as it decreases the overall strength, it can simultaneously serve to increase ductility due to the transformation-induced plasticity effect. Consequently, the influence of austenite can be advantageous or detrimental, depending on the precise application requirements [12].

In literature, studies have been carried out on the possibility of using a direct-aging heat treatment on M300 maraging steel processed by AM and to avoid the expensive and time-consuming solutioning step. The direct-aging behavior of L-PBF M300 has been investigated by Kim et al., who found that it is possible to harden the material up to its maximum hardness at a relatively low tempering temperature, i.e. 450°C. The effect of a direct aging heat treatment was compared with a more conventional solution treatment followed by aging and showed that the highest tensile strength can be achieved by direct aging [13]. Direct aging, in fact, promotes the precipitation of the Ni_3_(Mo, Ti) and Fe_2_Mo strengthening phase in the As Built (AB) martensitic matrix making it possible to achieve the desired hardness. Mutua et al. documented that the most substantial precipitation occurs under the DA condition. Avoiding solutioning, involves a significant reduction in elongation in the solution-aged state, but no noteworthy alterations in mechanical properties [14]. Tan et al. also compared the microstructure and mechanical properties of M300 processed by L-PBF in different post-processing states and showed that direct aging allows the achievement of hardness values similar to conventionally processed values [15]. Yin et al. [16] reported a systematic investigation on the optimization aging temperature and time for L-PBF M300 and showed that the maximum hardness is achieved after 3 h at 490°C. Finally, Moses et al. [17] proposed an in-depth investigation on the heat treatment effects on strength and fracture toughness demonstrating that a direct aging of 480°C for 6 h allows the achievement of the better mechanical performances.

To conclude, as also reported in the recent review by Guo et al., there is a limited body of literature that addresses the influence of process and post-process conditions on the microstructure and properties of this steel when processed using powder-based L-DED. This paper describes a comprehensive investigation into how process parameters and deposition atmosphere influence both the microstructure and mechanical properties of M300 processed through powder L-DED. Additionally, it delves into the consequences of Direct Aging Heat Treatment (DAHT) on these factors, thereby enhancing the understanding of the behavior of M300.

## 2. Materials and methods

**Figure 1.**
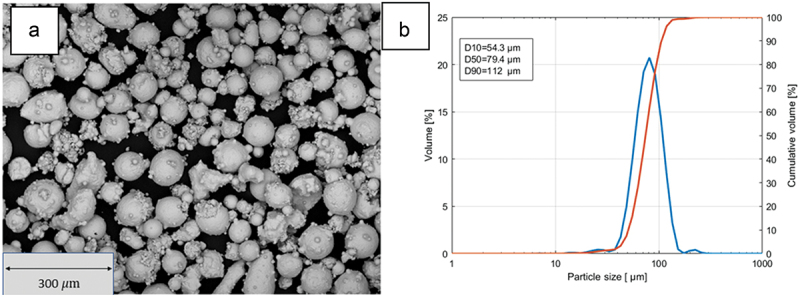
(a) SEM micrograph of the 18-Ni 300 powder, (b) particle size distribution of 18Ni-M300 powder.

A pre-alloyed 18-Ni 300 (M300) gas atomized powder with a particle size distribution in the range 45–106 supplied by Carpenter Additive (Carpenter Technology Corporation, PA USA), was used as raw material to produce all the samples. The chemical composition of the powder, as indicated in the conformity certification, is presented in Table 1. Figure 1(a) illustrates the overall morphology of the batch, aligning with DED standards. The particle size distribution was determined using a Malvern Panalytical Mastersizer 3000 (Malvern Panalytical ltd., UK) laser diffraction analyzer, revealing a median diameter (D50) of 79.4 μm. The diameter range between D10 and D90 was 53.4–112 μm (Figure 1(b)). The low-intensity peaks observed for equivalent diameters below 50 µm may be attributed to the presence of shells and satellites, visible in the SEM image, which, however, do not affect the flowability.

**Table 1.** Chemical composition (weight fraction, %) of the adopted 18-Ni 300 steel powder in accordance to ASTM A579 [18].

Ni	Mo	Co	Ti	C	Si	Mn	S	P	Al	Fe
18,00	4,7	8,6	0,8	0,3	0,1	0,10	<0,010	<0.010	0,08	Bal.

The experiments were performed on a demonstrator bench based on the Laserdyne 430 DED system of Prima Additive S.p.A. and, to verify the effect of the argon protective gas on the specimen properties, it was equipped also with the inert chamber optional accessory. This component essentially consists of a plexiglass cylinder mounted on the printing base with proper sealing, that supports a nylon membrane. The other end of the membrane is fixed with an appropriate housing on the deposition head, which remains bounded inside the realized room. The inertization was realized before the deposition with a purge cycle using the shielding gas supply. Since the chamber is not airtight, a pumping system is not required to avoid overpressure. The oxygen content was continuously monitored through a Servomex Servopro MultiExact Gas Analyzer (Servomex ltd., UK) mounted on the cylinder and a transducer that allows the continuous temperature monitoring inside the chamber during the argon depositions. The oxygen content was below 100 ppm during the entire Ar building processes. Two sets of process parameters in the material process window were selected, both in air and in argon. These parameters named Low Power (LP) and High Power (HP), were chosen as they have different energy density values (reported in Table 2) and provide an acceptable material densification. The nominal laser spot (*S*) was 2mm and the scanning strategy is bi-directional with 90° inter layer rotation. The energy density is defined as:

**Table 2.** Cubes building parameters and measured chamber temperature.

Sample code	Ed [J/mm^2^]	Deposition Environment	Chamber Temperature [°C]
LP Air	33.75	Air	∼25 °C
HP Air	33.45	Air	∼25 °C
LP Ar	33.75	Ar	∼300 °C
HP Ar	33.45	Ar	∼300 °C

$$E_{d}=\frac{P}{v_{s}*S};$$

Where P is the power and $v_{s}$ is the scanning speed.

The DED samples were cut along the building direction and polished according to a standard metallographic procedure. The relative density of the samples was evaluated with the Archimedes principle, following the test procedure described in ASTM B-311 [19]. Mass measurements were conducted using an analytical balance KERN ABS 80-4N (KERN & SOHN GmbH, Germany) having a resolution of 0.10 mg, equipped with the density measurement setup KERN YDB-03 (KERN & SOHN GmbH, Germany). Each sample underwent five measurements to obtain an average data. Moreover, the inherent porosity was assessed through cross-section observation using a LEICA DMI 5000 M (Leica Microsystems GmbH, Germany) Optical Microscope (OM). Data extraction involved statistical methods, selecting 30 images at various sample heights. The software ImageJ was employed to assess the distribution of the porosity within each sample.

To assess the sample thermal evolution, Differential Scanning Calorimetry (DSC) analyses were performed in Ar atmosphere with a heating rate of 20°C/min up to 1000°C using a TGA-DSC Setaram 92/16.18 (Setaram Instrumentation Inc., France). The M300 DED samples were post processed by means of DAHT keeping the samples at a temperature of 480°C for a duration of 6 hours, followed by furnace cooling.

All the specimens, both in the AB and DAHT conditions, were then etched using Marble etchant (a mixture consisting of 4 g of copper sulfate, every 20 ml of hydrochloric acid, and 20 ml of water). For the AB condition, the etching duration was 10 s, while for the DAHT condition, it was reduced to 5; the etching process for this steel is challenging, and immersion and swabbing were used together to achieve satisfactory results.

To analyze the microstructure morphology and distribution of precipitates, images were captured at varying magnifications using a Leica EZ4 W (Leica Microsystems GmbH, Germany) stereo microscope, an optical microscope (OM), a TESCAN s9000G (Tescan Group a.s., Czech Republic) field emission scanning electron microscopy (FESEM) with electron backscattered diffraction (EBSD), and a Thermo Fisher Phenom XL G2 (Fisher Scientific co., Ma USA) benchtop SEM with energy-dispersive X-ray detector (EDS) option. The EBSD analysis was conducted at 20 keV, 10 nA, with a step size of 0.55 μm. The primary cellular arm spacing (PCAS) was measured on the OM images using a proprietary image analysis toolbox developed in MATLAB, which utilized the triangle technique. PCAS values were used to determine the cooling rate values and to investigate the materials thermal history.

The phase content was determined by X-ray diffraction (XRD) analysis using a X-Pert Philips diffractometer (Malvern Panalytical ltd., UK) with Cu Kα radiation. The setup was operated at 40 kV and 40 mA with a step size of 0.013° over the 2θ range from 30 to 100°, and the X-Pert High Score software was used to deconvolute and integrate the XRD patterns. Finally, the mechanical behavior was evaluated by measuring the hardness of the samples using an ERNST NR3 (CISAM-ERNST s.r.l., Italy) Rockwell hardness tester with 150kg Rockwell C scale. Six measurements were taken at different sample heights, and the average value was recorded.

## 3. Results and discussion

**Figure 2.**
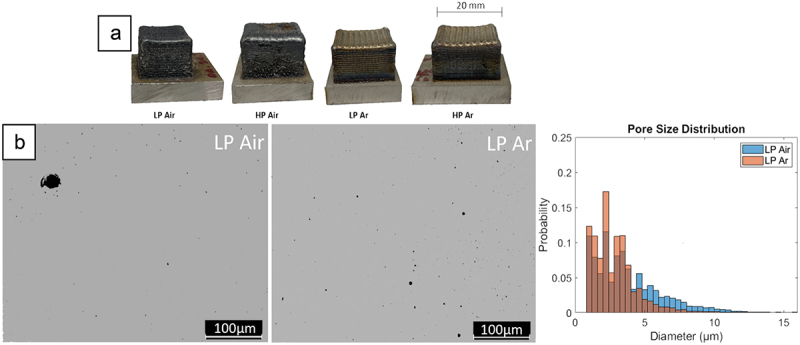
(a) AB DED M300 samples, (b) LP Air/LP Ar pores size distribution.

Upon preliminary visual examination, it was evident that samples built in air displayed an uneven surface with a fully developed oxide layer (Figure 2(a)). All the AB samples were characterized by comparable relative densities (Table 3), leading to the conclusion that the deposition atmosphere has no remarkable impact on densification. However, to better investigate the effect of the process atmosphere on the consolidation, a detailed cross-sectional examination was performed showing that in all the cases highly circular pores were observed, suggesting a connection to gas entrapment during the meltpool solidification. Furthermore, two distinct levels of pore sizes can be identified: air deposited samples feature a few defects with diameters exceeding 5 μm, approximately double the size of those found in inert depositions. Conversely, inert depositions demonstrate a consistently uniform distribution of microporosities (Figure 2(b)), indicating a more even dispersion compared to air deposited samples.

**Table 3.** Relative density of the samples.

	Rel. Density Archimede [%]	Rel. Density Imaging. [%]
LP Air	99,436 ± 0.005	99,766 ± 0.39
HP Air	99,369 ± 0.0005	99,893 ± 0.06
LP Ar	99,374 ± 0.004	98,798 ± 0.15
HP Ar	99,699 ± 0.004	99,553 ± 0.91

### 3.1. As-built microstructure

The microstructure of a DED processed steel is intricately tied to the thermal conditions experienced during its deposition. Consequently, it primarily results from the substantial thermal gradients and temperature fluctuations generated through the layers due to the intrinsic heat treatment (IHT) encountered during the process.

**Figure 3.**
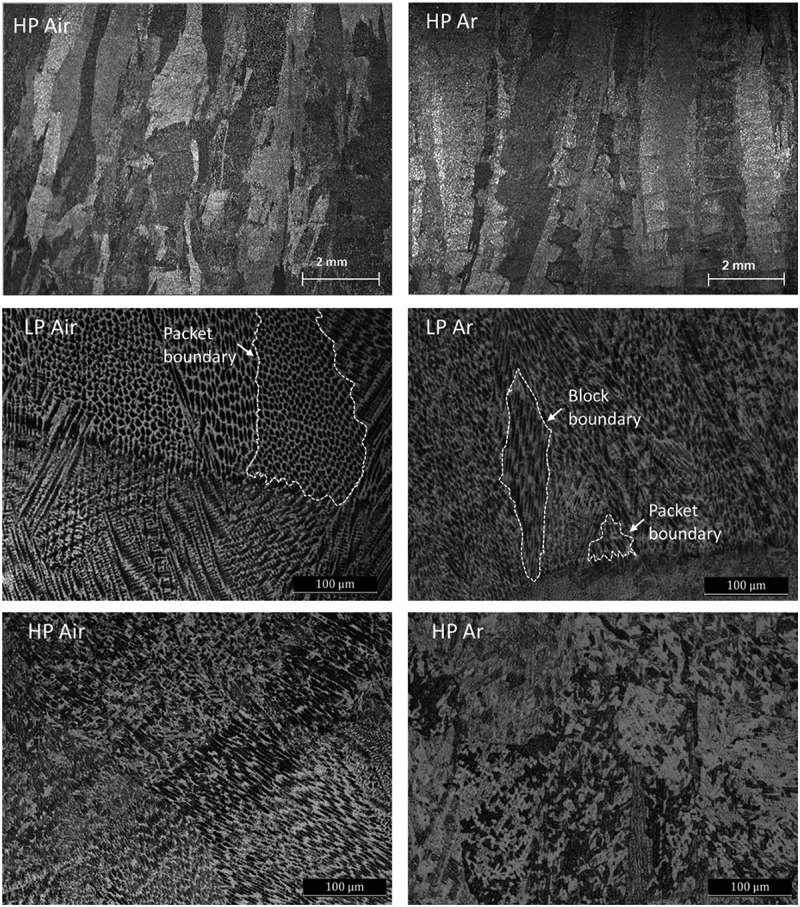
SM and OM images of XZ cross sections of LP air, HP air, LP Ar and HP Ar samples.

Figure 3 shows the characteristic morphologies of the vertical cross section of DED M300 samples at different level of magnification with both stereo- and optical-microscopes.

The low magnification images show that, in all cases, in the AB condition, coarse columnar prior γ-grains can be observed (Figure 3). These grains can grow across several layers thanks to the epitaxial growth mechanisms that arise due to the partial melt back of previously solidified layers [20].

Based on higher magnification OM observations, all samples also exhibit the characteristic microstructural pattern typically encountered in steels processed by DED. This pattern comprises interconnected molten pools where martensite blocks and packets containing either cellular or equiaxed dendritic structures coexist. A comparison between the microstructure of samples built in different conditions reveals that larger martensitic grains packets are formed in samples processed in air while elongated block grains prevail in Ar deposition (arrows and white dashed lines in Figure 3). This preliminary remark about the different grain morphology suggests that air samples solidify with a lower cooling rate.

**Figure 4.**
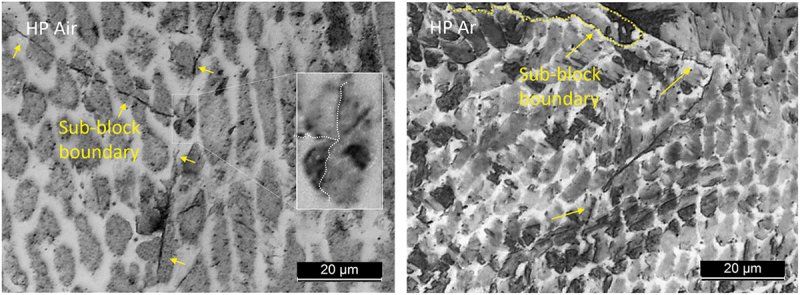
Martensite grain boundaries in OM images of HP air, HP Ar. Yellow dashed line indicates the block boundary while the yellow arrow indicate sub-block grain boundaries.

Given these considerations, a more detailed analysis of the relation between the cellular structures within packets/blocks was undertaken. Deeper magnification allows us to notice that the cells located in martensite packets exhibit a homogeneous appearance, while inside the martensite blocks there are sub-block structures that cross some of the cells (Figure 4). Similar observations were reported by Mei et al. in a L-PBF study [21]. Blocks and sub-blocks are the first martensite grains that form, and, with low cooling rate, they evolve in packets. These considerations also confirm the hypothesis of the higher cooling rate of Ar samples.

Moreover, at the meltpool boundaries, there is a prevalence of columnar dendrites while, in the central regions of the melt pools, equiaxed cellular microstructures tend to predominate. The morphology and the size of the dendritic microstructures are of great interest as it can be connected to different solidification aspects such as temperature gradient at the liquid/solid interface (G), solid/liquid interface travel rate (R), alloy composition, and undercooling degree. The aforementioned parameters can explain the solidification mode based on the calculation of the morphology parameter (G/R) and cooling rate level (G x R) [22,23]. Taking into account the observed morphologies, it can be said that towards the base of the molten pool, G attains its maximum value, while R tends to 0, resulting in a large G/R ratio, thus promoting a planar extension growth of the solidification structure. As it rises from the bottom of the melt pool, R gradually increases, causing a decrease in the G/R value. Consequently, a cellular dendritic structure evolves along the direction of layer stacking, which coincides with the direction of heat flow. Further towards the center of the melt pool, as G/R continues to decrease, the microstructure undergoes a transformation towards an equiaxed cellular configuration.

Preliminary examination of the images captured on the samples obtained in both atmospheric environments reveal the solidification phenomena that lead to this peculiar microstructure. It is well known that the heat input exponentially decreases moving away from the laser center. Consequently, in the peripheral areas, the molten material experiences a substantial undercooling, which promotes the initiation of grains containing elongated cells.

Furthermore, the internal cellular microstructure exhibits distinct size variations along the samples height, prompting a comprehensive investigation of the PCAS values to obtain a more complete view of the cooling rate experienced by the material under different building conditions. This also allowed us to calculate the cooling rate (C_r_), determined using the following equation [15]:

$$C_{r}={\left(\frac{\mathrm{PCAS}}{80}\right)}^{-\frac{1}{0.33}}$$

**Figure 5.**
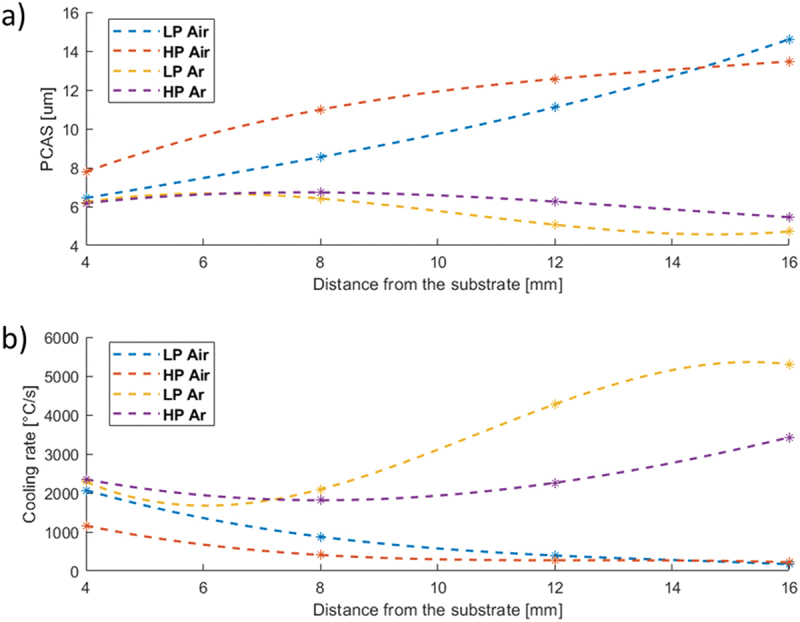
PCAS (a) – Cooling rate plots (b).

Figure 5 presents the trends extracted by a third-order interpolation as a function of the distance from the substrate. The graphical representations show a monotonic increase in PCAS values in the air-built samples. In contrast, in the case of Ar-built samples, a slightly decreasing trend is observed. The behaviors of cooling rate values, calculated according to the mentioned equation, are also reported in Figure 5. It is evident that, in the first layers the cooling rate is similar for all conditions (∼2000 °C/s) and that, in the Air environment, as the build progress, the cooling rate decreases, while it intensifies in the Ar atmosphere.

These results confirm the hypothesis about the different cooling rate based on the grain morphology observed by the optical microscope (Figure 3). In particular, these trends suggest that in free air depositions, heat dissipation primarily occurs toward the substrate: the cooling rate, in fact can be considered almost constant starting from 8 mm above the substrate (middle of the process), with a rate of about 450°C/s. While conduction is the only relevant mechanism during air deposition, inert chamber depositions require a more detailed description. In fact, the Ar environment processes are characterized by higher cooling rates and an increasing behavior to values of about 3000–4000°C/s. Such cooling rates are consistent with the ranges experimentally extracted by Jeong et al. [24] in a similar experimental setup.

It is hence evident that the different environmental conditions affect the heat transfer mechanisms. Argon is a gas frequently used for quenching processes due to its thermophysical properties. Its convective coefficient, in the 300–500°C temperature range of the inert chamber (Table 2), is fivefold greater than that of air at 25°C (hAr_300°C_ = 25 W/m^2^K; hAir_25°C_ = 5 W/m^2^K) [25]. Therefore, in the initial stages of Ar depositions, conduction emerges as the prevailing mechanism, similar to those in air due to the large temperature gradients existing between the molten pool and the cold substrate. As the deposition progresses, the substrate temperature increases and the importance of conduction, as a contributing factor, decreases, giving way to convection, which conversely gains importance along with the temperature rising.

A closer analysis of the curves also suggests that both LP cooling rate curves lay below their respective HP curves, indicating that the high power used during the DED process creates large melt pools and a large increase in build plate temperature, resulting in a lower cooling rate. This result is in line with previous data on similar materials [26,27].

### 3.2. As-built phase characterization

**Figure 6.**
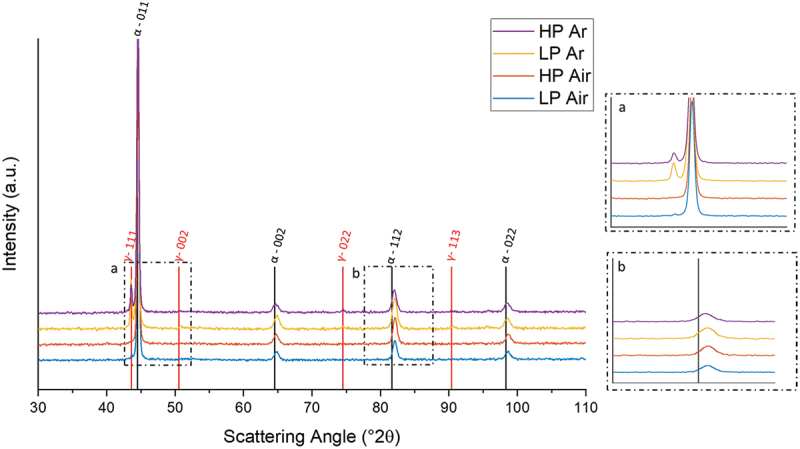
As-built XRD patterns.

X-ray diffraction analyses are useful in determining the phase content. Figure 6 summarizes the AB XRD patterns of samples built in all processing conditions. The samples predominantly exhibit a martensitic structure, as indicated by shifted α-Fe peaks (Figure 6 – inset b). A closer look to the spectra also reveals the presence of low intensity γ-Fe peaks in both Ar conditions and in the HP Air one. This result could be surprising considering that, in traditional processing, a fully martensitic structure is achieved even when the material solidifies with low cooling rates [4].

**Figure 7.**
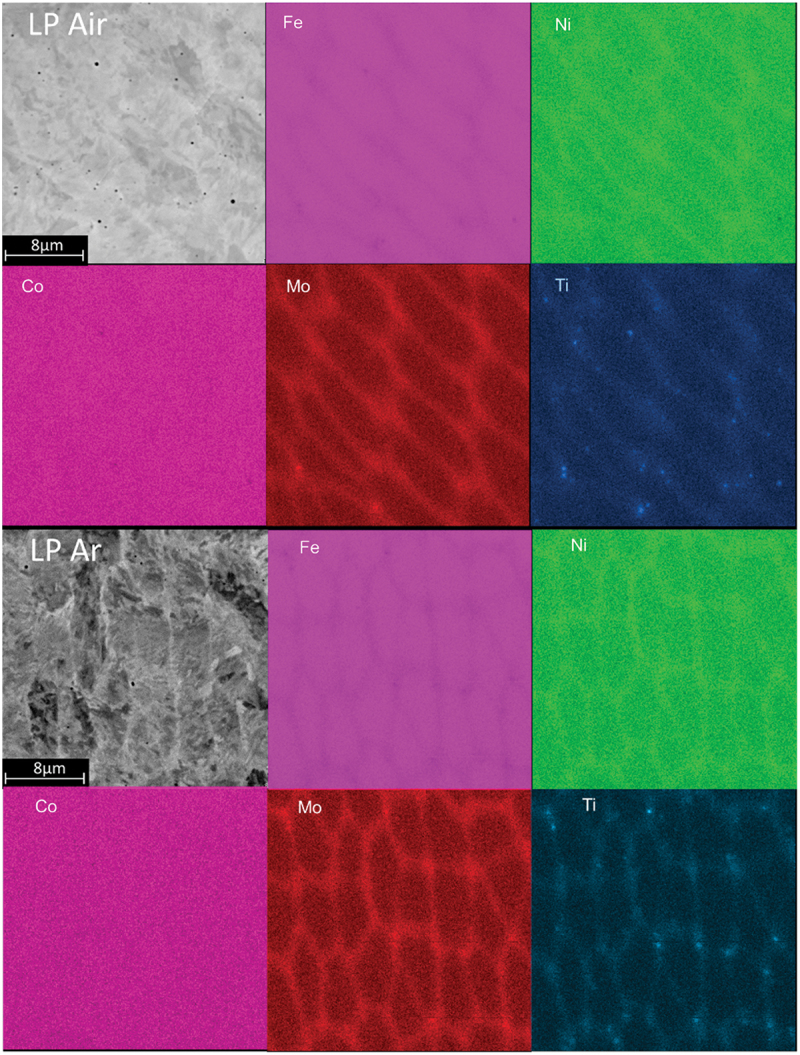
AB LP-Air, LP-Ar EDS map chosen as representative.

In order to understand the reasons for the presence austenite of in the AB state, EDS and EBSD analyses were carried out. The EDS maps indicated segregation of Ni, Mo, and Ti at the cell boundaries (Figure 7). This segregation is significantly greater in the Ar environment. Additionally, the maps clearly reveal numerous Ti-rich particles, mostly evident in the air-exposed sample. These particles are likely to be Ti oxides resulting in a strong Ti oxidation tendency [28]. No significant distinctions can be observed between the HP and LP configurations for each environmental condition based on all conducted surveys.

**Figure 8.**
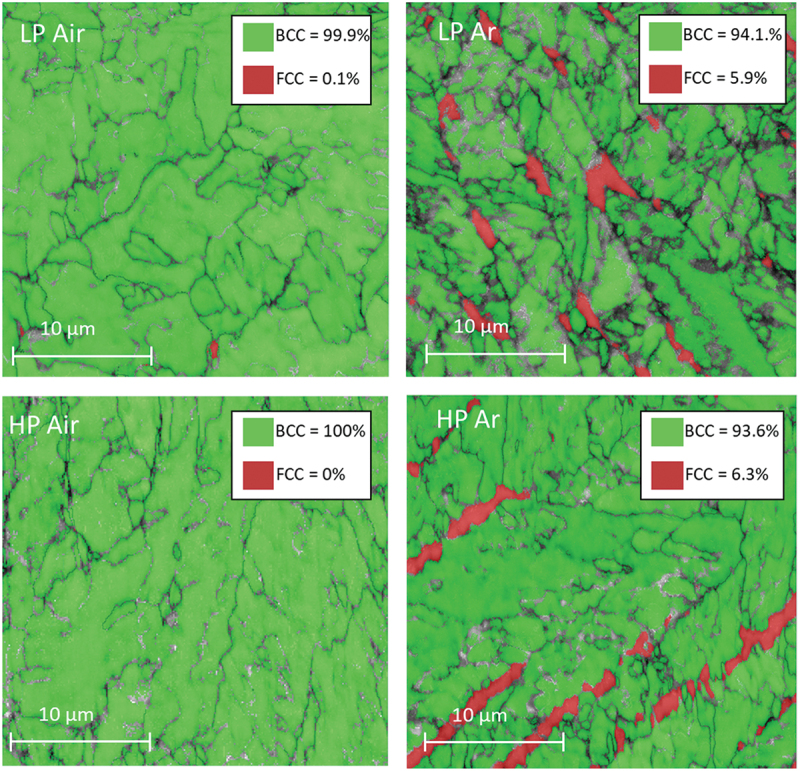
As built EBSD phase maps.

EBDS was employed to draw phase maps based on the crystallographic information. In all the conditions the results coming from the XRD patterns were confirmed (Figure 8). The microstructure is characterized by a martensitic matrix ($\alpha^{\prime }$) where traces of γ-Fe phase are located essentially at the boundaries of the martensitic cells.

Figure 8 presents, also, the amount of austenite in AB condition. The quantification of austenite was performed as area fraction in the EBSD phase maps images. The data align with the intensity of the austenite peaks observed in the X-ray diffraction patterns (Figure 6).

These analyses allowed the understanding of the solidification mechanisms that lead to the presence of a certain austenite quantity in DED-processed M300 steel. It is well-known that two types of γ-phases can be identified in maraging steels: retained and reversed austenite. Retained austenite is formed during cooling from the $A_{3}$ temperature down to the martensitic start temperature (Ms), while reversed austenite is formed due to diffusion-controlled mechanisms [29]. In this material, both retained and reversed austenite occur for different reasons. First, austenite is retained during cooling from $A_{3}$ despite the very high cooling rate. This can be explained considering the cellular microstructure that solidifies during DED, which is characterized, as evidenced by EDS (Figure 6), by the segregation of elements such as Ni, Ti and Mo in the interdentridic regions. Although these types of constituents are well known α stabilizers, when used as alloying elements in extra low carbon steels, their segregation causes a reduction in Ms temperature as described by many empirical relations [30,31]. Therefore, the central part of the cell transforms first and generate stresses that hinders the martensitic transformation of the cell boundaries [32].

Furthermore, it’s worth noting that Ms is strongly influenced by grain size. In other words, the finer the microstructure, the lower the martensite start temperature. Therefore, in AM-produced parts, the fine microstructure also contributes to the retention of austenite [33,34]. Finally, some of the austenite could also be due to the reversion phenomenon due to the IHT.

The higher austenite content in both Ar samples with respect to the Air ones might seem surprising considering that air is mainly constituted by nitrogen which is a strong γ-stabilizer (Figure 6). Based on the previous considerations, the different γ content in samples processed in Ar and Air can once again be attributed to the substantial divergence in thermal history encountered by the samples and to the resulting different cell size (Figure 5). The high cooling rate of the Ar process results in deeper microstructural refinements, which leads to a greater reduction in the Ms temperature and a consequent stronger retention of austenite [35].

Clearly, the presence of austenite in the AB condition can affect the mechanical properties of the steel. Specifically, it does not undergo hardening during subsequent aging heat treatments, while concurrently enhancing ductility [36]. Finally, in the context of LP Air, where the low energy density causes smaller molten pools with respect to the high power one, the attained cooling rates are higher causing a higher retention of austenite in the microstructure.

### 3.3. Thermal evolution

**Figure 9.**
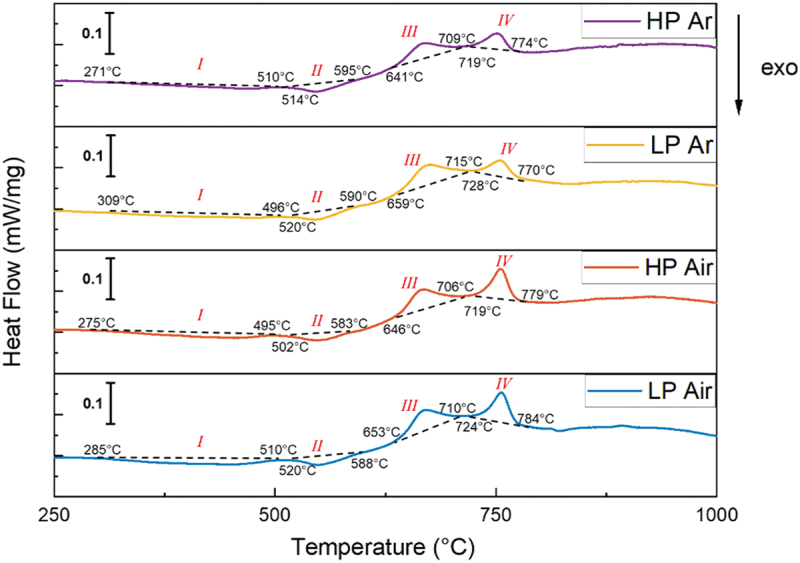
DSC patterns.

To study the phase transformation behavior of the AB specimens and select the most appropriate aging temperature, DSC analyses were performed on the samples built in different conditions (Figure 9). The results showed that all the DSC curves had two exothermic peaks (I and II) followed by two endothermic peaks (III and IV).

In particular, peak I is due to the recovery of martensite, to the precipitation of carbides and coherent strengthening phases. In this range of temperature, a minor hardening is realized with the formation of coherent intermetallic compounds [37,38]. Peak IIis due to grain growth, and the formation of the incoherent strengthening precipitates [38–40] while peak III was associated with the transformation from martensite to austenite [41–43]. Finally, peak IVwas attributed again to the martensite reversion, and the dissolution of precipitates or recrystallization [41,42,44,45].

The plots clearly illustrate a significant contrast in the intensities of the first endothermic peak between the two environmental conditions. This discrepancy can be justified considering the deposition temperature of the inert chamber, which promotes a consistent amount of precipitation during processing.

In reference to the third and fourth peaks (III, IV), the higher intensity observed in air deposition can be attributed to the nearly complete martensitic transformation that takes place under these processing conditions. This conclusion is readily apparent in the XRD patterns (Figure 6), where it is challenging to distinguish austenitic peaks.

Considering these DSC results, the temperature of 480°C was selected for DAHT in the present study. This temperature is suitable for adequate aging as it is just above the first precipitation temperature (Peak I) and before Peak II, which causes the precipitation of detrimental incoherent strengthening phases [15,46,47].

### 3.4. Heat treated microstructure

**Figure 10.**
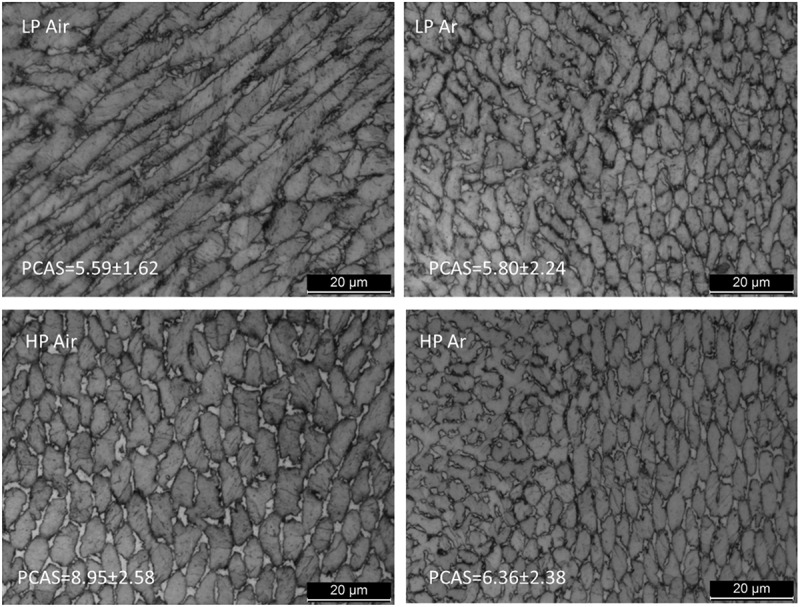
Heat treated OM micrographs.

**Figure 11.**
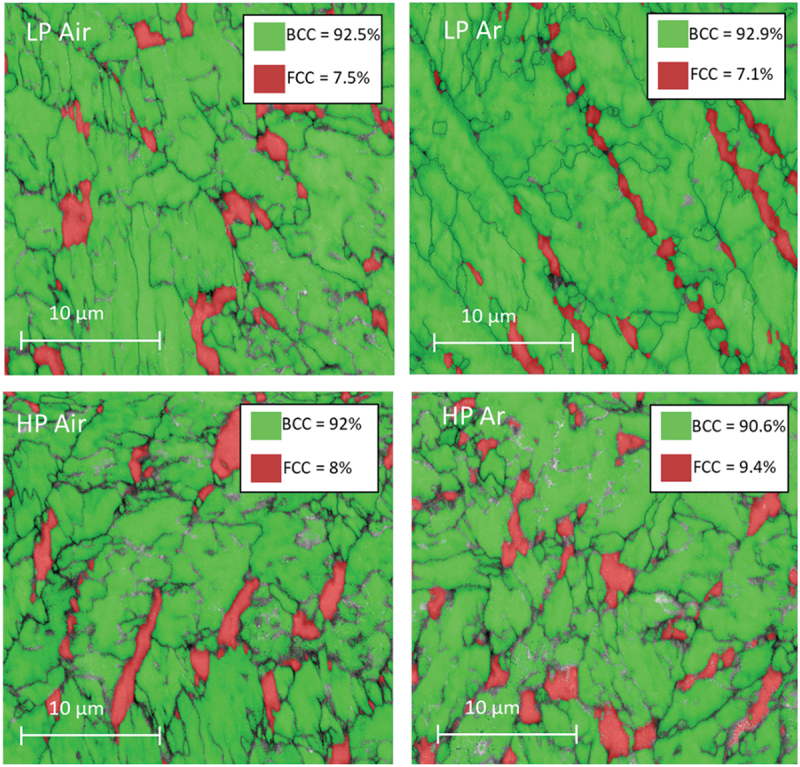
DAHT EBSD phase maps.

Figure 10 illustrates the OM micrographs of the material after DAHT, revealing that the material maintained a cellular microstructure. The PCAS was evaluated as in the AB case, and no trends along the build direction could be extracted. The mean PCAS values are around 6 µm. It is interesting to highlight that the cells of both samples from air deposition underwent a refinement if Figures 5 and 10 are compared. Moreover, the emergence of a distinct phase along cells boundaries is more evident. This second phase was characterized using EBSD, establishing that it is austenite (Figure 11). All DAHT samples contain similar amount of austenite always higher than their AB counterpart (Figure 8). The increase in the γ-Fe content from the as built condition is strictly related to the reversion phenomenon that arises during the aging treatment [40]. In Ar samples, the reversion arises mainly where austenite was retained because this growth pattern eliminates the necessity for nucleation, and it is thus energetically advantageous. On the contrary, in Air samples, where retainment did not happen during the DED process, the reverted austenite forms in different locations. In these cases, the reverted austenite may nucleate preferentially at the boundary of martensite packets and block/sub -block. As shown in Figure 4, these boundaries often cross the cells; therefore, this reversion phenomenon is responsible for the cells size refinement observed in the case of Air samples [48,49].

**Figure 12.**
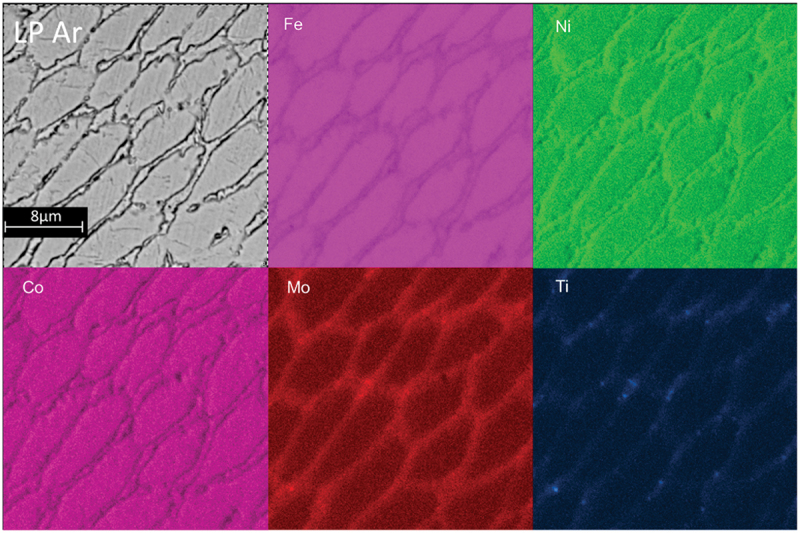
DAHT LP Ar EDS map.

Heat treated specimens were also analyzed by EDS. The map results are presented in Figure 12. Clearly, during the heat treatment, diffusion and precipitation phenomena take place as evident from the different distribution of alloying elements with respect to the AB state (see Figure 6).

In particular, the microstructural analysis revealed variations in the enrichment and depletion patterns at different cell boundaries. In the core of the cells, there is a notable enrichment of Co while, moving to the cell border, enrichments in Mo, Ti, Ni are observed, coupled with a depletion in cobalt. The intercellular regions, instead, exhibit enrichments in Mo and Co, with a slight increase in titanium.

Comparing the DAHT maps with the AB ones (Figures 7 and 12, respectively), it can be noticed that the DAHT influences the distribution of alloying elements. Cobalt, for examples, was homogeneously distributed in the AB state and diffused during heat treatments toward the cell core and the intercellular regions. Co does not actively participate in the age-hardening reaction, but it serves the purpose of reducing the solubility of Mo within the martensitic matrix. This, in turn, promotes the precipitation of Ni_3_Mo which has a favourable lattice compatibility with the martensitic matrix.

Along the cell borders, the high Ni-Mo-content, can be attributed to the metastable Ni_3_Mo phase, while the Ni-Ti enrichments, may be due to the rapid reactivity of titanium with nickel, likely leading to the possible formation of the Ni_3_Ti phase during aging.

Finally, in the intercellular region, the presence of Co together with Fe and Mo suggests the presence of the stable Fe_2_Mo precipitates which transform from the metastable Ni_3_Mo in extended aging [47]. Similarly, to the AB case, also XRD analysis (here not reported) were performed confirming what extracted by EDS and EBSD.

### 3.5. Mechanical analysis

**Figure 13.**
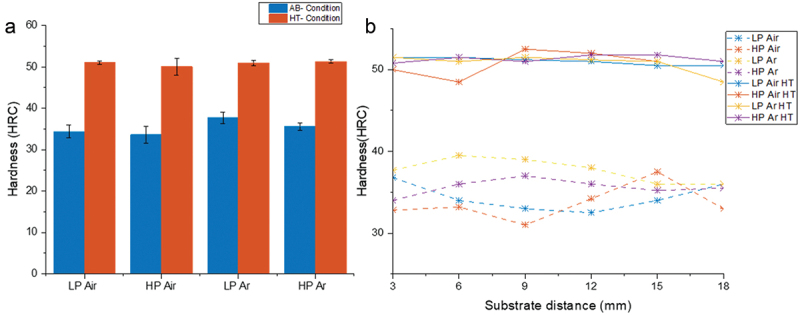
(a) Mean samples hardness values; (b) hardness trends along samples height.

The measured hardness values are summarized in Figure 13(a). The AB specimens exhibit hardness values within the range of 34–37 HRC. In contrast, the hardness is significantly enhanced, reaching 50–51 HRC after the direct aging heat treatment. The lower HRC values in AB condition can be attributed to the existence of a relatively soft martensitic phase. In maraging steels, in fact, the martensite is a soft iron-nickel phase, constituted by a solid solution of nickel (with minor quantities of Co and Mo) within α-Fe [47]. This martensite imparts a significant level of ductility in contrast to the conventional carbon-based solid solutions martensite encountered in traditional steels.

DAHT results in an increase in HRC through the operation of two distinct mechanisms: precipitation strengthening and strain strengthening. Precipitation during aging is e.g. by the high dislocation density of martensite that allows space for the precipitates [40]. The primary hardening effect is brought about by the needle-shape Ni_3_Ti precipitates [50], which induce a distortion effect within the matrix. This distortion, in turn, interacts with dislocations, thereby enhancing strain strengthening [51]. Comparable mechanical performances values were obtained by Jägle et al. on L-PBF samples after a similar heat treatment including solutioning step [12]. Moreover, the measured hardness aligns precisely with the ranges for traditionally manufactured parts. Wrought components register a mean hardness value of 35 HRC that rises up to 54 HRC after the heat treatment [52,53].

Figure 13(b) illustrates the hardness patterns across the vertical cross-sections of the AB and DAHT specimens. Notably, the AB state exhibits divergent trends in hardness in comparison to the PCAS profiles seen in Figure 5(a). Specifically, the HRC values of the Air samples exhibit a mild upward trend, while those in the argon environment demonstrate a slight decrease. This suggests a correlation between hardness values and the cooling rate. In contrast, the DAHT samples consistently maintain a constant hardness level along their height which is in line with the constant PCAS value observed in Figure 10. As a result, it can be deduced that a finer cellular distribution corresponds to higher hardness values.

## 4. Conclusions

In the present study, 18Ni-300 samples were fabricated by direct energy deposition and the effects of process parameters, deposition environment and post-process conditions on the material microstructure and properties were investigated. The following conclusions can be drawn:

- Dense M300 maraging steels samples could be successfully produced by DED using both Air and Ar atmosphere and with both laser power values used in this study.
- The differences in environmental conditions influence the material microstructure as they affect the heat transfer mechanisms. Conduction is prevalent in the initial stages of argon depositions due to large temperature gradients. As the deposition progresses convection becomes more significant, particularly as the chamber temperature rises. In air deposition, on the contrary, conductive phenomena dominate the entire process.
- In AB conditions, different content of retained austenite was found in the samples. These different austenite contents were associated to the different cooling conditions experienced by the samples.
- The AB hardness values observed align with the cooling rate and the consequent microstructural evolution, showing that high hardness values are correlated to low cell size.
- The effect of different processing conditions is eliminated by the post-processing heat treatment. DAHT causes the increase of the austenite content thanks to the reversion mechanism which promotes the growth of the γ regions at the cell boundaries. Similar austenite contents were detected in all DAHT conditions.
- Aging also increased the hardness and allowed the achievement of constant hardness values along the sample height confirming that the effect of the thermal history is eliminated.

## References

[1] F42 Committee. Terminology for additive manufacturing technologies [Internet]. ASTM International; [cited 2023 Nov 30]. Available from: http://www.astm.org/cgi-bin/resolver.cgi?F2792-12A

[2] Saboori A, Aversa A, Bosio F, et al. An investigation on the effect of powder recycling on the microstructure and mechanical properties of AISI 316L produced by directed energy deposition. Mater Sci Eng A. 2019;766:138360. doi: 10.1016/j.msea.2019.138360

[3] Liang D-M, Zhu Y-Z, Liu G-H. Development and application of maraging steels. Jinshu RechuliHeat Treat Met. 2010;35:34–16.

[4] ASM International Handbook Committee. ASM handbook. Volume 1, properties and selection: irons, steels, and high-performance alloys [Internet]. 10th ed. Materials Park (OH): ASM International Materials Park; 1990. doi: 10.31399/asm.hb.v01.9781627081610

[5] Guo L, Zhang L, Andersson J, et al. Additive manufacturing of 18% nickel maraging steels: defect, structure and mechanical properties: a review. J Mater Sci Technol. 2022;120:227–252. doi: 10.1016/j.jmst.2021.10.056

[6] Rońda N, Grzelak K, Polański M, et al. The influence of layer thickness on the microstructure and mechanical properties of M300 maraging steel additively manufactured by LENS® technology. Materials. 2022;15(2):603. doi: 10.3390/ma15020603 35057320 PMC8780661

[7] Yao Y, Huang Y, Chen B, et al. Influence of processing parameters and heat treatment on the mechanical properties of 18Ni300 manufactured by laser based directed energy deposition. Opt Laser Technol. 2018;105:171–179. doi: 10.1016/j.optlastec.2018.03.011

[8] Chen B, Huang Y, Gu T, et al. Investigation on the process and microstructure evolution during direct laser metal deposition of 18Ni300. Rapid Prototyp J. 2018;24:9. doi: 10.1108/RPJ-01-2018-0022

[9] Félix-Martínez C, Ibarra-Medina J, Fernández-Benavides DA, et al. Effect of the parametric optimization and heat-treatment on the 18Ni-300 maraging steel microstructural properties manufactured by directed energy deposition. Int J Adv Manuf Technol. 2021;115(11–12):3999–4020. doi: 10.1007/s00170-021-07320-y

[10] Bai Y, Wang D, Yang Y, et al. Effect of heat treatment on the microstructure and mechanical properties of maraging steel by selective laser melting. Mater Sci Eng A. 2019;760:105–117. doi: 10.1016/j.msea.2019.05.115

[11] Laleh M, Sadeghi E, Revilla RI, et al. Heat treatment for metal additive manufacturing. Prog Mater Sci. 2023;133:101051. doi: 10.1016/j.pmatsci.2022.101051

[12] Jägle EA, Choi P-P, Van Humbeeck J, et al. Precipitation and austenite reversion behavior of a maraging steel produced by selective laser melting. J Mater Res. 2014;29(17):2072–2079. doi: 10.1557/jmr.2014.204

[13] Kim D, Kim T, Ha K, et al. Effect of heat treatment condition on microstructural and mechanical anisotropies of selective laser melted maraging 18Ni-300 steel. Metals [Internet]. 2020;10(3):410. doi: 10.3390/met10030410

[14] Mutua J, Nakata S, Onda T, et al. Optimization of selective laser melting parameters and influence of post heat treatment on microstructure and mechanical properties of maraging steel. Mater Des. 2018;139:486–497. doi: 10.1016/j.matdes.2017.11.042

[15] Tan C, Zhou K, Kuang M, et al. Microstructural characterization and properties of selective laser melted maraging steel with different build directions. Sci Technol Adv Mater. 2018;19(1):746–758. doi: 10.1080/14686996.2018.1527645

[16] Yin S, Chen C, Yan X, et al. The influence of aging temperature and aging time on the mechanical and tribological properties of selective laser melted maraging 18Ni-300 steel. Addit Manuf. 2018;22:592–600. doi: 10.1016/j.addma.2018.06.005

[17] Paul MJ, Muniandy Y, Kruzic JJ, et al. Effect of heat treatment on the strength and fracture resistance of a laser powder bed fusion-processed 18Ni-300 maraging steel. Mater Sci Eng A. 2022;844:143167. doi: 10.1016/j.msea.2022.143167

[18] TBT Committee. Standard specification for superstrenght alloy steel forgings [Internet]. [cited 2024 Mar 28]. Available from: https://compass.astm.org/document/?contentCode=ASTM%7CA0579_A0579M-20%7Cen-US&proxycl=https%3A%2F%2Fsecure.astm.org&fromLogin=true

[19] B09 Committee. Test method for density of powder metallurgy (PM) materials containing less than two percent porosity [Internet]. ASTM International; [cited 2024 Jan 31]. Available from: http://www.astm.org/cgi-bin/resolver.cgi?B311-22

[20] Basak A, Das S. Epitaxy and microstructure evolution in metal additive manufacturing. Ann Rev Mater Res. 2016;46(1):125–149. doi: 10.1146/annurev-matsci-070115-031728

[21] Mei X, Yan Y, Fu H, et al. Effect of aging temperature on microstructure evolution and strengthening behavior of L-PBF 18Ni(300) maraging steel. Addit Manuf. 2022;58:103071. doi: 10.1016/j.addma.2022.103071

[22] Selcuk C. Laser metal deposition for powder metallurgy parts. Powder Metall. 2011;54(2):94–99. doi: 10.1179/174329011X12977874589924

[23] Saboori A, Gallo D, Biamino S, et al. An overview of additive manufacturing of Titanium components by directed energy deposition: microstructure and mechanical properties. Appl Sci. 2017;7(9):883. doi: 10.3390/app7090883

[24] Jeong J, Webster S, Liao S, et al. Cooling rate measurement in directed energy deposition using photodiode-based planck thermometry (PDPT). Addit Manuf Lett. 2022;3:100101. doi: 10.1016/j.addlet.2022.100101

[25] Narazaki M, Kogawara M, Qin M, et al. Measurement and database construction of heat transfer coefficients of gas quenching. J Mech Eng. 2009;55:167–173.

[26] Montero-Sistiaga ML, Godino-Martinez M, Boschmans K, et al. Microstructure evolution of 316L produced by HP-SLM (high power selective laser melting). Addit Manuf. 2018;23:402–410. doi: 10.1016/j.addma.2018.08.028

[27] Larimian T, Kannan M, Grzesiak D, et al. Effect of energy density and scanning strategy on densification, microstructure and mechanical properties of 316L stainless steel processed via selective laser melting. Mater Sci Eng A. 2020;770:138455. doi: 10.1016/j.msea.2019.138455

[28] Springer H, Baron C, Szczepaniak A, et al. Efficient additive manufacturing production of oxide- and nitride-dispersion-strengthened materials through atmospheric reactions in liquid metal deposition. Mater Des. 2016;111:60–69. doi: 10.1016/j.matdes.2016.08.084

[29] Xu X, Ganguly S, Ding J, et al. Microstructural evolution and mechanical properties of maraging steel produced by wire + arc additive manufacture process. Mater Charact. 2018;143:152–162. doi: 10.1016/j.matchar.2017.12.002

[30] Liu C, Zhao Z, Northwood DO, et al. A new empirical formula for the calculation of MS temperatures in pure iron and super-low carbon alloy steels. J Mater Process Technol. 2001;113(1–3):556–562. doi: 10.1016/S0924-0136(01)00625-2

[31] Jägle E, Sheng Z, Kürnsteiner P, et al. Comparison of maraging steel micro- and nanostructure produced conventionally and by laser additive manufacturing. Materials. 2016;10(1):8. doi: 10.3390/ma10010008 28772369 PMC5344583

[32] Herzog D, Seyda V, Wycisk E, et al. Additive manufacturing of metals. Acta Mater. 2016;117:371–392. doi: 10.1016/j.actamat.2016.07.019

[33] García-Junceda A, Capdevila C, Caballero FG, et al. Dependence of martensite start temperature on fine austenite grain size. Scr Mater. 2008;58(2):134–137. doi: 10.1016/j.scriptamat.2007.09.017

[34] Funch CV, Christiansen TL, Somers MAJ. Gaseous nitriding of additively manufactured maraging steel; nitriding kinetics and microstructure evolution. Surf Coat Technol. 2022;432:128055. doi: 10.1016/j.surfcoat.2021.128055

[35] Król M, Snopiński P, Hajnyš J, et al. Selective laser melting of 18NI-300 maraging steel. Materials. 2020;13(19):4268. doi: 10.3390/ma13194268 32992702 PMC7579195

[36] Lang FH, Kenyon N. Welding of maraging steels. Sudbury (CA): International Nickel Company; 1971.

[37] Decker RF, Peters DT, Cupp CR. Source book on maraging steels: a comprehensive collection of outstanding articles from the periodical and reference literature. Materials Park (OH): American Society for Metals; 1979.

[38] Bui N, Dabosi F. Contribution to the study of the effect of molybdenum on the ageing kinetics of maraging steels. Cobalt. 1972;57:192–201.

[39] Decker RF, Goldberg A. Source book on maraging steels: a comprehensive collection of outstanding articles from the periodical and reference literature. Materials Park (OH): American Society for Metals; 1979.

[40] Pereloma EV, Shekhter A, Miller MK, et al. Ageing behaviour of an Fe–20Ni–1.8Mn–1.6Ti–0.59Al (wt%) maraging alloy: clustering, precipitation and hardening. Acta Mater. 2004;52(19):5589–5602. doi: 10.1016/j.actamat.2004.08.018

[41] Guo Z, Sha W, Li D. Quantification of phase transformation kinetics of 18 wt.% Ni C250 maraging steel. Mater Sci Eng A. 2004;373(1–2):10–20. doi: 10.1016/j.msea.2004.01.040

[42] Menapace C, Lonardelli I, Molinari A. Phase transformation in a nanostructured M300 maraging steel obtained by SPS of mechanically alloyed powders. J Therm Anal Calorim. 2010;101(3):815–821. doi: 10.1007/s10973-010-0745-5

[43] Goldberg A, O’Connor DG. Influence of heating rate on transformations in an 18 per cent nickel maraging steel. Nature. 1967;213(5072):170–171. doi: 10.1038/213170a0

[44] Saul G, Robertson JA, Adair AM. Source book on maraging steels: a comprehensive collection of outstanding articles from the periodical and reference literature. American Society for Metals; 1979.

[45] Bai Y, Zhao C, Zhang J, et al. Abnormal thermal expansion behaviour and phase transition of laser powder bed fusion maraging steel with different thermal histories during continuous heating. Addit Manuf. 2022;53:102712. doi: 10.1016/j.addma.2022.102712

[46] Casati R, Lemke J, Tuissi A, et al. Aging behaviour and mechanical performance of 18-Ni 300 steel processed by selective laser melting. Metals. 2016;6(9):218. doi: 10.3390/met6090218

[47] Tan C, Zhou K, Ma W, et al. Microstructural evolution, nanoprecipitation behavior and mechanical properties of selective laser melted high-performance grade 300 maraging steel. Mater Des. 2017;134:23–34. doi: 10.1016/j.matdes.2017.08.026

[48] Conde FF, Escobar JD, Oliveira JP, et al. Austenite reversion kinetics and stability during tempering of an additively manufactured maraging 300 steel. Addit Manuf. 2019;29:100804. doi: 10.1016/j.addma.2019.100804

[49] Zhang X, Miyamoto G, Toji Y, et al. Orientation of austenite reverted from martensite in Fe-2Mn-1.5Si-0.3C alloy. Acta Mater. 2018;144:601–612. doi: 10.1016/j.actamat.2017.11.003

[50] Vasudevan VK, Kim SJ, Wayman CM. Precipitation reactions and strengthening behavior in 18 Wt Pct nickel maraging steels. Metall Trans A. 1990;21(10):2655–2668. doi: 10.1007/BF02646061

[51] Kelly PM. The effect of particle shape on dispersion hardening. Scr Metall. 1972;6(8):647–656. doi: 10.1016/0036-9748(72)90120-2

[52] Kempen K, Yasa E, Thijs L, et al. Microstructure and mechanical properties of selective laser melted 18Ni-300 steel. Phys Procedia. 2011;12:255–263. doi: 10.1016/j.phpro.2011.03.033

[53] Standard SAE. AMS 6514H. Steel maraging bars forg tubing rings. 2012;18:8.

